# “We are like lemmings”: making sense of the cultural meaning(s) of suicide among the indigenous Sami in Sweden

**DOI:** 10.3402/ijch.v74.27669

**Published:** 2015-09-01

**Authors:** Jon Petter A Stoor, Niclas Kaiser, Lars Jacobsson, Ellinor Salander Renberg, Anne Silviken

**Affiliations:** 1 Sami Norwegian National Advisory Board on Mental Health and Substance Abuse (SANKS), Finnmark Hospital Trust, Karasjok, Norway; 2 Department of Psychology, Umeå University, Umeå, Sweden; 3 Division of Psychiatry, Department of Clinical Sciences, Umeå University, Umeå, Sweden; 4 Centre for Sami Health Research, Department of Community Medicine, UiT – The Arctic University of Norway, Tromsø, Norway

**Keywords:** Sami, suicide, indigenous, identity, Sweden, qualitative study

## Abstract

**Background:**

Suicide is a widespread problem among indigenous people residing in the circumpolar Arctic. Though the situation among the indigenous Sami in northern Scandinavia is better than among some other indigenous people, suicide is still regarded as a major public health issue. To adapt prevention strategies that are culturally attuned one must understand how suicide is understood within context. That is, the cultural meaning(s) of suicide.

**Objective:**

To explore and make sense of the cultural meaning(s) of suicide among Sami in Sweden.

**Design:**

Open-ended focus group discussions (FGDs) on the topic “suicide among Sami” were carried out in 5 Sami communities in Sweden, with in total 22 strategically selected Sami participants. FGDs were recorded, transcribed verbatim and analyzed through employing content analysis.

**Results:**

From the FGDs 4 themes emerged including “The Sami are fighting for their culture and the herders are in the middle of the fight,” “Suicide as a consequence of Sami losing (or having lost) their identity,” “A wildfire in the Sami world” and “Difficult to get help as a Sami.”

**Conclusions:**

Findings indicate that Sami in Sweden make sense of suicide in relation to power and identity within a threatened Sami cultural context. Suicide is then understood as an act that takes place and makes sense to others when a Sami no longer has the power to maintain a Sami identity, resulting in being disconnected from the Sami world and placed in an existential void where suicide is a solution. The findings are useful in development of culturally attuned suicide prevention among Sami in Sweden.

Indigenous people residing in the circumpolar Arctic have been found to account for some of the highest suicide rates worldwide. However, within the Arctic region there is great variance both within and across indigenous populations in different territories and contexts. Very high suicide rates have been reported from indigenous peoples in Russia (1), Alaska (2), Canada (3) and Greenland (4) while suicide rates among the indigenous Sami in Scandinavia are moderate (5). However, there are some striking commonalities with the situation in other parts of the Arctic, including elevated rates in comparison with majority populations (in the Sami case in comparison with Norwegians, Swedes and Finns), high suicide rates among young men, suicide clusters and use of highly lethal methods such as firearms and hanging (5, 6). Also, the negative impacts of suicide seem to be higher in Sami communities than majority society (as compared to rural and urban populations in Sweden), with higher prevalence's of exposure to suicidal expressions among Sami youth (7) and reindeer herding Sami (8).

Suicide is strongly related to mental health disorders, but researchers are also stressing the importance of understanding suicide among indigenous people in the Arctic in the context of long-lasting (9) and on-going (10) colonialization processes, including rapid social changes resulting in the breaking of traditional indigenous structures and insufficient social development. Indeed, some researchers partly reject the western psychiatric perspective and claim that “aboriginal suicide is different” (11), and that a different framework therefore has to be used to make sense of the phenomenon. In *Sápmi* (land of the Sami), Kaiser (12), Omma (13) and Silviken (14) have all argued that cultural context in general, and the difficulties of maintaining traditional livelihoods such as reindeer herding in particular, is crucial to understanding suicidal behaviour among Sami.

Hjelmeland and Knizek (15) have argued that qualitative methodology must be employed to understand cultural meaning(s) of suicide in particular contexts, and focus group discussions (FGDs) have been used as a culturally attuned way of investigating this (16, 17), also among indigenous people in the Arctic (10). Acknowledging the need for better understanding of indigenous ways of making sense of suicide to make prevention culturally attuned and relevant to the indigenous community (18), the present study aims to explore and make sense of the cultural meaning(s) of suicide among Sami in Sweden.

## Method

The researchers were aware, from personal experience, that suicide among Sami was frequently talked about in different Sami contexts in Sweden, and wanted to explore such talk in search for the cultural meaning(s) of suicide among Sami. To do so, the study employed open-ended FGDs (without interview guide and no topics introduced by the researchers) in which the participants (PTs) could set their own agenda, self-regulate and be in control of the narrative they individually and as a group created – eschewing and highlighting aspects of their choice. This approach assumedly made sure that topics covered in the FGDs were reflective of normative narratives regarding suicide among Sami in Sweden. The collected material was then explored in search of patterns of meaning (19), employing content analysis (20). The study followed the principles of the Helsinki declaration and was approved by the regional ethical committee at Umeå University, Sweden. In addition, although there are no specific ethical guidelines in Sweden for Sami research, the study paid attention to ensure a culturally safe process overall.

### Sample

Altogether 22 Sami PTs were recruited by the first author using a snow-ball method. They were strategically chosen based on recommendations and dialogue with Sami organizations and utilization of the private network of the first author, himself a Sami. To ensure dynamic FGDs and a rich dataset, care was taken to recruit PTs that were diverse in terms of: Sami identity (North-, Lule-, Ume- and Southsami), Sami language competency, work experience (traditional livelihoods, health care professionals, culture workers and academics among others), sex (which proved difficult, ending up with 6 male and 16 female PTs), age (ranging from 18 to 63 years of age) and experience(s) of suicide among Sami.

### Procedure

Five FGDs, varying in length between 1.5 and 2 hours, were held during spring 2012 on different locations in Swedish *Sápmi*. PTs were informed of aims and intentions of the study, including that participation might trigger strong emotions and that the researchers (of whom the second author was an experienced clinical psychologist) were prepared to deal with this. Informed consent was given prior to FGD. PTs were not financially compensated, except for travel expenses and a meal before or after (then combined with snacks before) the FGD. Encouraged by the researchers, all groups opted to engage in voluntary confidentiality agreements in regard to each other's narratives. The PTs were also encouraged to talk freely, as they would have in a “normal” group conversation, before initiating the discussions with the phrase “When talking about suicide among Sami, what you think is most important that we talk about?” The FGDs were led by either the first or second author (with the other taking notes and presenting a summary for the group to comment upon before ending) whose main priority was to support the participants voices through clarifying answers, asking for other opinions and making sure that all PTs were heard. The FGDs were audio taped and transcribed verbatim by the first author as soon as possible after the FGDs (within days, prior to the next FGD). The transcripts (de-identified to ensure the anonymity of the PTs) resulted in a data corpus of 122 pages (12 point, single-spaced).

### Analysis

The analysis phase overlapped data collection, with the researchers reflecting on their experiences directly after each FGD and the first author taking notes during transcription (in-between FGDs). Those notes were used to create a preliminary understanding of topics that seemed important, which allowed the researchers to better navigate the next FGD; aiming at gaining a more full understanding of topics already talked about (in previous FGDs) as well as looking out for new perspectives and/or topics. When all material was transcribed the first and second author conducted a content analysis guided by Graneheim and Lundman's (20) stepwise model, with the whole data corpus (from all FGDs) being collapsed into a single dataset. The purpose of the analysis was to inductively and systematically explore the material in search of re-emerging patterns of meaning that could be said to reflect meanings of suicide among the PTs, and then define, demarcate and describe those patterns.

1. To get an overview, the whole material was read through several times by the first and second author.
2. Units of meaning were identified and extracted into excerpts by the first author.
3. The excerpts were shortened (“condensed”) by the first author, while trying to keep the original meaning intact. The second author independently did the same thing with a smaller portion of the excerpts and then compared those with the first author's, to ensure consistency.
4. The shortened excerpts were turned into codes, based on interpretations of the latent content. The coding was carried out by the first author who repeatedly discussed interpretations with the second author (who read the material), followed by adjustments and re-coding (hence trying to ensure plausibility in interpretations). A portion of the excerpts was labelled “miscellaneous” because of the researchers’ inability to meaningfully interpret those excerpts in relation to the aims of the study.
5. The codes were abstracted and categorized into themes by the first author, then discussed between the first and second author. This was a process that included several re-categorizations (moving back and forth between codes and themes), as well as reading of the original transcripts to ensure that the themes were reflective of the content as a whole.
6. The other authors read the material and provided a critical discussion of it, resulting in excluding some themes relating to the PTs experiences of participating in the FGDs (deemed to be outside the scope of the article) and redefining (refining and demarcating borders and relations between) other themes.
7. The findings have been presented (by the first author) on numerous occasions (at least 10 times with Sami audiences) since 2012, resulting in a great deal of feedback and in-depth discussion with a wide range of Sami people (audiences including, for example, Sami mental health care personnel and groups of young male and female herders attending suicide prevention initiatives). Even though not part of the original methodology, these interactions have created a cumulative process of continuously re-viewing the findings in light of these experiences, resulting in a more refined understanding of the results at hand.

## Results

From the analysis emerged 4 themes including “The Sami are fighting for their culture and the herders are in the middle of the fight,” “Suicide as a consequence of Sami losing (or having lost) their identity,” “A wildfire in the Sami world” and “Difficult to get help as a Sami.” The themes are presented (below), with quotes from FGDs (using codes to identify individual PTs; the first letter indicating focus groups from A to E), to illustrate certain aspects.

### The Sami are fighting for their culture and the herders are in the middle of the fight

A central topic in the FGDs was that Sami are engaged in a fight to maintain Sami identity and culture, which was described as threatened by the surrounding society, and sometimes also from within. The outside threats included exploitation of Sami lands (by forestry, tourism, mining activities, wind- and hydro power plants and societal infrastructure), conflicts with authorities and local communities that often involved what was perceived as discrimination, including derogatory speech and/or actions. Also, specific threats to reindeer herding were described, such as weather conditions (including global warming and negative effects on grazing lands), amounts of predators on the land and legislation that makes it very hard to start as a new herder or come back again if you, or your father, have left. Inside threats included Sami not respecting each other's identity as authentic (e.g. due to lack of language competency and/or association to traditional livelihoods) and conflicts among herders due to herding being a zero-sum game (if one herder increases his herd, the others must reduce). Even though some PTs pointed out that being part of these conflicts were not always healthy, it was described that being Sami was to continue fighting for Sami identity despite the hardships, as illustrated by this change of opinions between 2 PTs:

B3: I think that the Sami; that we are a very resilient people. That is, we're used to many hardships and we don't give up easily.

B5: are we resilient or are we foolhardy? [Laughter].

In this context of conflict, reindeer herding was described as something of a stronghold for Sami culture. To live as a herder was understood as the best way to preserve Sami identity and be able to pass the legacy on to new generations but, perhaps paradoxically, also to be standing in the middle of the Sami fight, sometimes even being trapped within it:

B4: The reindeer herders are trapped in a cultural prison. (…) They are entrapped in their culture.

B2: I don't know if I agree with you. A prison, in my opinion, is a place I can't escape from. And I live in my culture, and I do not want to leave – so therefore it is not a prison.

This particular discussion (above) went on to conclude that Sami are like lemmings, meaning that they will continue to fight for their identity, regardless of their chances.^1^

### Suicide as a consequence of Sami losing (or having lost) their identity

The PTs associated the topic “suicide among Sami” to actual suicide cases among young Sami men who were living within, or associated with, reindeer herding. It was also said that those who had died in suicide were often regarded as joyful persons, that others didn't think had had suicidal wishes or plans. To this a bereaved friend said:

D2: He was absolutely not a person you saw as depressed or anything (…) it didn't show! Suddenly one day he was just gone.

Empathy was expressed in regard to those who had died, and the act of suicide was described as understandable in light of the existential predicament of a herder who was in danger of losing, or had lost, the means or power to continue life as a Sami. As another herder put it:

E4: I can somehow understand it – the reasoning [behind it] (…) what is the alternative here in life? (…) if it was my life and I did not have it [the herding] anymore – why shouldn't I cross over?

Suicide was thus framed within normality in this cultural context, as a way to avoid the existential void that would have been the (perceived) consequence of life without reindeer herding. A young man said it like this:

E1: [the suicides] are strongly associated with reindeer-herding (…) and with identity, also. (…) if you are faced with a choice between to abandon your own identity – that is your life really – to do something else, maybe there won't be much left of yourself?

### A wildfire in the Sami world

The PTs referred to a positive experience of belonging to a small, close knit Sami community, even when the “community” spanned over great geographical distances where Sami sometimes lived far apart from each other. The Sami network was described as “multiplex,” with each person having several different connections to the same other person (through family relations, work colleagues, friendship and membership in the same community), with a great many (Sami) connections in total. It was described that when a suicide occurs within the network, it becomes activated and over-flooded with communication about the suicide. A participant described it like this:

B2: You live in this network; you talk to these people (…) when something happens in a Sami community, it's like wildfire between the communities about what has happened.

To be part of the network at such times was described as overwhelming and very emotional, including feelings of surprise, anger, fear and being without control. Another PT, a father, expressed his worry about it like this:

C4: It almost became a mass-hysteria among young Sami (…) on Facebook it took enormous proportions. (…) It was like a grassfire that only spread (…) and many became extremely affected by that.

The implicit fear that suicide would spread through contagion within the network was also explicitly expressed by others:

A4: It is almost as if it was contagious!

### Difficult to get help as a Sami

In the FGDs, it was said that the Swedish health care system was not fit to help struggling Sami. PTs had heard of Sami who had sought out mental health care, but then experienced their Sami identity as an obstacle both related to cultural values among Sami (possibly meaning that Sami should not talk about mental health problems), and the lack of Sami cultural knowledge among health care personnel. Worry was expressed that Sami will not seek help when they need it, or that they will have to explain and defend their culture and lifestyle before they can get help. Sami were described to be used to that kind of explaining and defending, though not in the help-seeking context but from conflicts with authorities and non-Sami locals, and it was described as something difficult and challenging. A PT said:

A5: [you're] afraid that you won't be understood in the Swedish care system. It is hard to talk about reindeer herding or the Sami culture.

## Discussion

The aim of this article is to explore and make sense of the cultural meaning(s) of suicide among Sami in Sweden. First, we note that there is relatively little contradiction within the results. While this could be due to several reasons, including a selection bias or relative unanimity among the Sami in regard to this issue, we believe the most conceivable explanation is the presence of a single prevailing narrative, so strong that it overshadows other narratives. We suggest that the symbolic image imbedded in “We're like lemmings” might be regarded as a central part of that narrative, including that Sami, just like lemmings, are perceived to be fighting for survival but often face defeat due to power inequality.

**Fig. 1:**
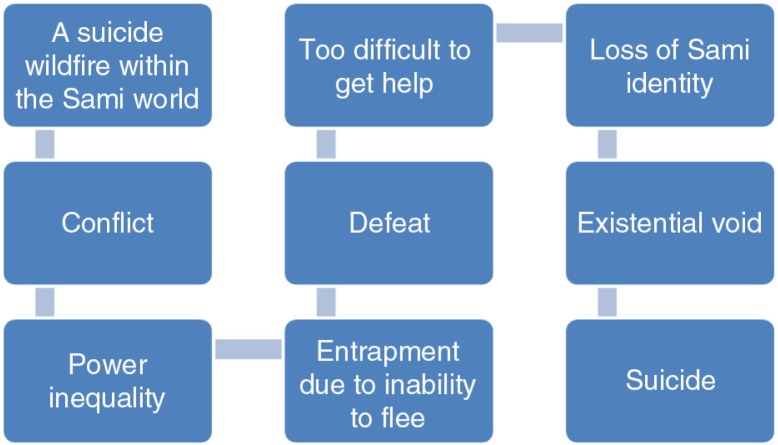
Illustration of a prevailing narrative on suicide among Sami, as interpreted by the authors in relation to the results.

**Fig. 2:**
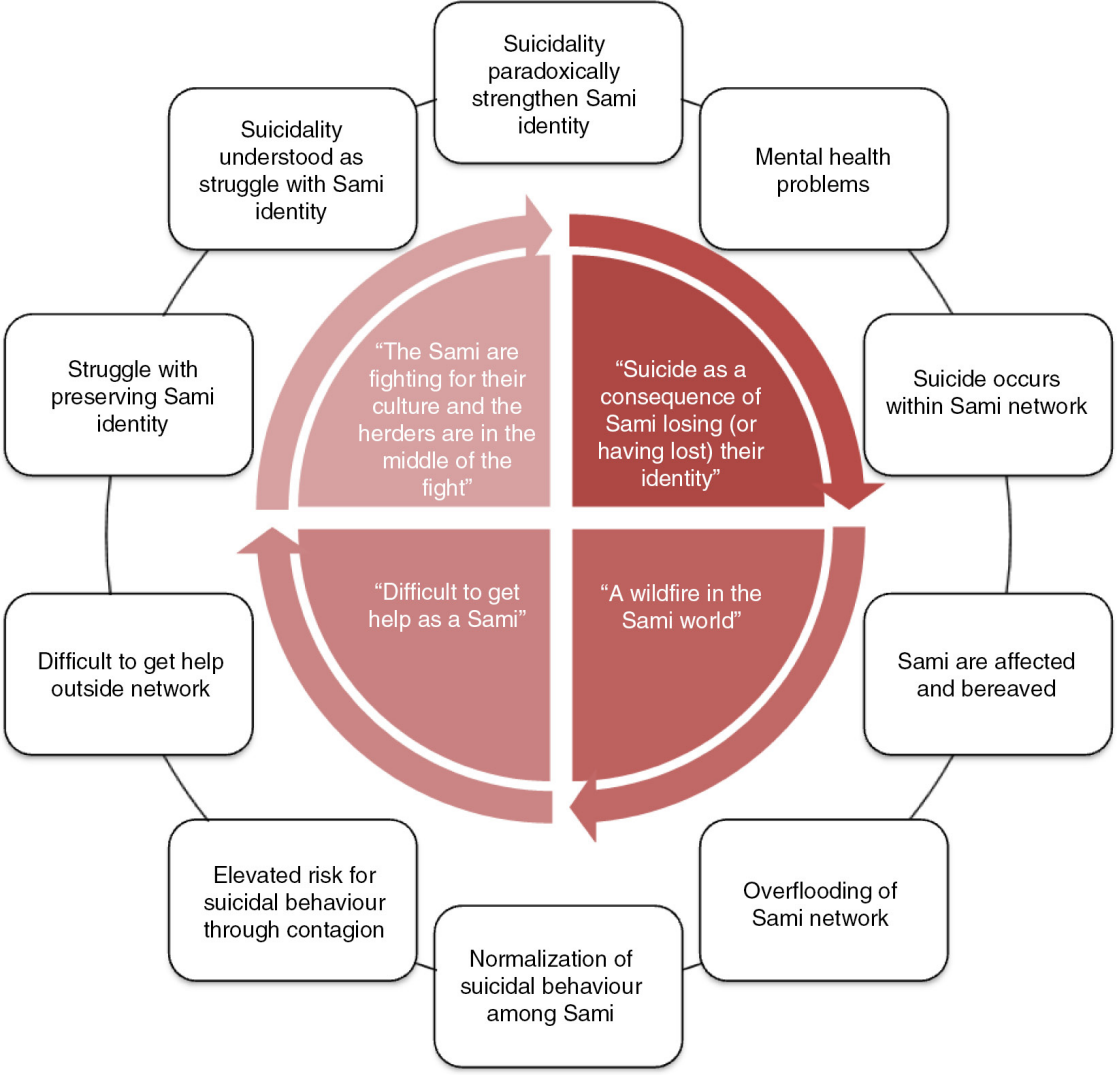
Theoretical model of meanings (inside the circle) relating to suicide among Sami in Sweden, and socio-cultural processes (outside) possibly underlying those meanings.

In Fig. 1, we give an illustration of how this narrative might provide an understanding of suicide, as an act that takes place and makes sense to others when a Sami become entrapped in (due to being unable to back down from) a conflict, but no longer has the power to maintain a Sami identity, resulting in being disconnected (from the Sami world) and placed in an existential void where suicide is a solution. This highlights that suicide is framed by the Sami participants as acts within the socio-cultural normality (thus excluding suicide as acts of “crazy” people), affecting individuals engaged in a fight for their Sami identity. A key feature of the lemmings’ analogy is that lemmings do not die by their own hand but from their inability to flee in face of death. That is, in this perspective, suicide is a consequence of the threats from majority society against the Sami culture. We argue that these converging meanings of identity and power in relation to suicide (as acts in a political context, yet within normality) make up a very problematic socio-cultural dynamic wherein engaging in suicidal behaviour, or even dying by suicide, can paradoxically *strengthen* Sami identity. To the extent that this narrative is part of the socio-cultural context in which struggling Sami individuals navigate their actions, those individuals might chose to engage in suicidal behaviour to be part of the social narrative placing them inside the Sami world rather than, for example, talk openly about their *individual* mental health issues (and risking experiencing oneself as disconnected from the Sami world due to breaking a cultural taboo). In Fig. 2, we seek to theoretically illustrate what socio-cultural processes (the outer circle) we believe might be underlying the themes that emerged in this study.

While the problems formulated within the themes could be regarded as shared experiences to a degree where they become socially accepted as normative, they are not necessarily “true” in the objective sense, but rather perceived to be. However, many of the issues raised also make sense in light of previous research. For example, there is little doubt that reindeer herding is very central to Sami identity (21, 22), that this life is seriously threatened and that this combination causes mental health problems and contributes to suicidality among reindeer herders (8, 12, 13, 22). Also, the close-knit Sami world has been previously proposed as a possible factor behind suicide clusters and contagion effects among Sami (6–8). Furthermore, low confidence in (Swedish) health care among Sami reindeer herders is documented and has been interpreted as a hindrance for full enjoyment of such services (23). Also, at least some of the elements in the “We're like lemmings” narrative can fit rather well within mainstream models of suicidality, as can be illustrated through examining the narrative in light of the “Interpersonal–Psychological Theory of Attempted and Completed Suicide” (IPT-ACS) and it's key features; *thwarted belongingness*, *perceived burdensomeness* and *acquired capability*, as proposed by Joiner (24). The *perceived burdensomeness* (perceiving yourself as a burden to others or the society) described in the IPT-ACS can be related to the Lemmings-narrative as an internalized perception deriving from the Swedish mainstream society disregarding Sami value, for example expressed through exploitations of Sami land without consent, and failure to address Sami needs for culturally attuned health care – areas where Sweden have received international critique from United Nations officials (25). The *thwarted belongingness* (perceiving yourself as having lost prior established meaningful relationships) can be interpreted in conjunction to the existential void (loss of Sami identity and connectedness to the Sami world) perceived to be the result of individual defeat due to lack of power to continue the fight for Sami identity. Furthermore, the prerequisite of an *acquired capability* to enact lethal self-injury is very present in the image of Sami as lemmings, maybe it is even the key feature of it, since lemmings are known for their capability to be fearless in face of death. This also makes sense in light of traditional Sami upbringing values aiming at youngsters becoming autonomous, self-determined andhardened (26), which paired with the reindeer herders extensive experience of killing reindeers (traditionally done with a precisely aimed knife stick) and other animals (with rifle) might have habituated them to not fearing death or bodily harm – and hence acquire the capability to inflict lethal self-injury.

### Limitations

First, it is important to note that this is a qualitative study that explores the cultural meaning(s) of suicide, not the suicides *per se*. Furthermore, there are some other serious limitations to this study, including the small number of participants (ruling out generalizing findings, even among Sami in Sweden), the high risk for selection bias (in spite of actions taken to recruit PTs that were diverse) due to the snow-ball recruitment method, based in part on utilization of the private network of the first author, as well as the very high likelihood that PTs eschewed topics during data collection. Another limitation is that all FGDs were held in Swedish, likely resulting in difficulties for PTs with other mother tongue (Sami) and discussions not being able to fully tap into cultural understandings which might be imbedded within, and not translatable from, Sami language. In fact, it is likely that another study design, with other PTs and researchers, would have ended up with different results, or at least made other interpretations of similar results.

However, the limitations were hard to avoid and should be considered in relation to the study's aim, including investigating a potentially sensitive (for some, certainly taboo) subject within an indigenous group with well-grounded historical reasons to mistrust the academic society in general and health researchers in particular (for example, due to “scientists” taking skull measurements to prove Sami racial inferiority during the first half of the 20th century) (27). Furthermore, researchers have described how Sami often face numerous obstacles when talking about health problems with (non-Sami) professional personnel, including having to overcome Sami cultural norms of (not) talking about such problems (28) and navigating a complex trans-cultural arena when doing so (29). In light of this, the first author being an *insider* was something of a prerequisite for ensuring a culturally safe research process, including reducing potential power inequalities between academic researchers and indigenous participants as well as striking a line between respecting Sami traditions and scientific demand (for example, done through taking time to get to know each other, including sharing a meal and revealing kinships, before continuing to the FGDs). Furthermore, the very intention of choosing open-ended FGDs was to provide PTs with a possibility to assume agency (thereby reducing the influence of the researchers and the risk of contaminating data), and thus be able to highlight and eschew the discussions as they saw fit; freely creating a narrative reflective of their cultural meanings of suicide. From our perspective, the strive for trustworthiness and ethically sound decision-making converged in this study, and all things considered we propose that the results are indeed reflective of some – *though not necessarily all* – key features of the cultural meanings of suicide among Sami in Sweden. The extent to which these results might be transferable is a question for the reader to answer, but it is advised that any attempt to transfer and interpret these results in other contexts should be done jointly, and cautiously, between indigenous knowledge holders and academic scholars.

### Future research

In general, perhaps with the exception of suicide rates (5, 6), we still have limited scientific knowledge regarding suicide among Sami. At this point we propose that further research be conducted in 2 specific directions. First, in-depth investigation of actual suicide cases could help shed light on the suicidal processes leading up to suicide among Sami, for example whether this process includes specific cultural and/or contextual features of importance (which this study implies that it does). Second, exploring and/or developing culturally attuned suicide prevention initiatives carry a potential to reduce suicide rates among Sami. In regard to the latter, we would support strengthening “pan-Sami” border crossing collaborations on suicide prevention and establishing closer links between researchers, Sami communities (especially youth organizations) and governmental institutions responsible for decision making and delivering of health care services in *Sápmi*.

## Conclusions

This study adds to, and complements, previous research on suicide among Sami through providing in-depth exploration of the cultural meanings of suicide among Sami in Sweden (as understood and talked about by Sami). The resulting narrative of “Sami as lemmings” highlights issues of power inequality and identity struggle in relation to suicide. In the discussion, we show that while these understandings also makes sense in light of previous research and a mainstream model of suicidality, they can possibly increase suicidality among Sami as struggling individuals might strengthen their (threatened) identity through enacting (Sami) suicidality. We propose that suicide prevention among Sami should take this into account to make best use of the preventive potential. After all, “suicide only becomes possible insofar as it is imaginable, insofar as it is meaningful, insofar as one can make sense of it, whether as a decision, as a last resort, or as a statement of desperation” (16, p. 2089).

## Footnotes

1 The lemming, found in mountain and tundra areas throughout Sápmi, is known among Sami for its fiery behaviour, never fleeing when threatened, often placing them in harm's way.
